# Artificial Respiration in Chloroform Collapse

**Published:** 1894-04-28

**Authors:** Benjamin Ward Richardson


### Artificial Respiration in Chloroform Collapse.

By Sir Benjamin Ward Richardson, M.D., F.R.S.

In my last paper on this subject I pointed out the risks that may arise from the use of galvanism or other kind of electrical excitation in chloroform collapse. I proceed to indicate what seems to me the only rational methods that can be carried out in order to produce recovery.

First and foremost, then, artificial respiration must be placed. It is not necessary in saying this to assume that death under chloroform purely occurs from failure of respiration, because there is always some primary failure of the heart, which does not mean actual arrest in the motion of the heart. The fact that after death by chloroform the heart is usually found in movement in the lower animals has led to the idea, natural enough in itself, that the organ is still performing vital functions, and that, therefore, death must occur from failure of the respiration. There is, however, a deception on this point. Mere rythmical motions of the auricles and ventricles of the heart does not mean necessarily vital motion. I have noticed that the heart has three grades of motion. (*a*) Full vital motion of propulsion, such as is going on while the body is alive and perfectly conscious. (*b*) Partial vital motion of propulsion, such as is going on while the body is alive, breathing, but unconscious; conditions of syncope, catalepsy, and anasthesia. (*c*) Passive motion of auricles and ventricles without propulsion, and with arrested respiration, or what may be called respiratory death; the motion of collapse, most commonly presented under chloroform collapse.

It is this third condition of the heart and of respiratory death we have to deal with under chloroform collapse. In this state the heart itself is reduced in volume, that is to say, it is contracted and contains very little blood. It is as if it were passing into rigor mortis, and, in fact, when it does not come back to its natural action, it does pass into rigor mortis, which means its completer arrest, and, as a natural consequence, death of itself and of the whole body, which in the normal state it feeds with the vital blood. That the muscles of respiration have failed in their function during this state is due indeed to the circumstance that they are not receiving from the heart their necessary portion of blood, and therefore fail in their working power. Meanwhile the lungs, owing to the passive contraction of their elastic structure, which continues independently of blood supply, become resistant and to a certain extent collapsed. Also the elastic resistance of the pulmonary artery progresses, and opposition is therewith made to entrance of blood into the lung, an opposition which the enfeebled heart has little power to overcome. This opposition is exceedingly great, as any experimentalist may prove by endeavouring with a syringe fixed in the pulmonary artery to force a current over the lesser circulation.

These facts lie at the root of all endeavours to start the lesser circulation in chloroform collapse. If we do not understand them we are easily led to suppose that the whole difficulty lies in the failure of the respiratory current, whereas the failure is, at all events, partly, in the heart itself, and may be so primarily, although the organ immediately after death may appear to be alive. It might be supposed that if this were true, artificial respiration would never of itself bring back life, but I have seen in the most demonstrative manner that it may do so.

In watching the motions of the heart in the third degree (above described) while the process of artificial respiration was being carried on, I have seen the heart pass into the second degree, and then into the first, with almost immediate recovery of vital muscular movements throughout the body, and with recovery of attempted respiratory movements. The phenomena are most remarkable, and have to be seen actually to be properly understood. There is, as it were, a leap of cardiac motion from the third or feeblest stage into the second, or, as I would call it, the syncopal or cataleptic. In this second stage the heart increases in volume, and there is a feeble pulsation in the pulmonary artery. Then there is a second leap, in which the auricles and ventricles become suddenly tense with blood. The heart enlarges to twice the size which it appeared during the third degree. The pulmonary artery distends and pulsates strongly; the lungs become charged with blood; the left heart fills; the aorta begins to pulsate strongly and actively; all the muscles receive their new supply of blood; respiratory movements are made active; and, in a word, vitality is restored. Towards this resuscitation artificial respiration is the only means at present at our command, and that it may prove successful experiment most decidedly illustrates. In the lower animal I have seen it succeed as long as seven minutes after all external evidences of life had ceased, but its success or failure depends upon varying circumstances. Thus it is a very great obstacle to its success for the temperature of the day to be very high, for under that condition there is quick coagulation of blood, and therewith plugging of the pulmonary circuit. On the other side, extreme cold is an obstacle, the best temperature being about 60 deg. on Fahrenheit's scale. Again, any rough motion of the body is an obstacle, for under such the little remaining motion there is of the heart, and which has to be encouraged into a more active motion, is most easily arrested, and then all is over. Lastly, too violent artificial respiration is itself an obstacle, for when the lungs are vigorously and rapidly inflated the very pressure which they make on the heart in the closed thorax is sufficient to stop the feeble cardiac pulsation.

It follows from these observations that the ordinary methods of artificial respiration by Sylvester's or the Marshall Hall plans, or by any other that causes active movement of the body externally must be injurious, as tending to reduce the power of the feebly moving heart. What is really wanted is the quick, gentle emptying of the lungs of the air containing the chloroformwhich they hold, and the gentle refilling of the chest with new air introduced without undue pressure, so that it may reach the feeble current of blood which may possibly be making its way over the pulmonary circuit.

In the absence of apparatus of any kind mouth-to-mouth insufflation is the best method that can be devised. By fixing an indiarubber tube to the nostril of the lower animal, and by emptying and filling the chest of the animal in this way, but very gently, recovery from deepest collapse can almost always be effected at a medium temperature of the air. It does not appear that the presence of carbonic acid in the breath of the operator does any material amount of injury, and there is always exhaled by the breath a sufficient quantity of oxygen to restart the vital respiration. The objection lies in the personal act of setting up breath-to-breath respiratory action, and I have endeavoured to meet this objection by constructing the double-acting hand-bellows which bear my name. These bellows, made of rubber, exhaust by one bulb and fill by another, so that by the same motion of the hand the chest of the narcotised body is emptied and refilled. Should these bellows not be at hand the ordinary bellows with a single bulb answer fairly well, or a pair of parlour bellows answer. The method consists in placing a nozzle from such bellows in one of the nostrils, closing the other nostril by pressure over the tube, closing the mouth, then expanding the bellows so as to exhaust the chest, or by gently movement of compression, to fill the chest. It is not at all necessary to follow natural movements of respiration, in so far as number is concerned. Indeed, the gentler expiration and inspiration are carried out the better, eight to ten exhausts and inflations per minute being sufficiently adequate.

